# Do Brain-Derived Neurotrophic Factor Genetic Polymorphisms Modulate the Efficacy of Motor Cortex Plasticity Induced by Non-invasive Brain Stimulation? A Systematic Review

**DOI:** 10.3389/fnhum.2021.742373

**Published:** 2021-09-28

**Authors:** Ryoki Sasaki, Sho Kojima, Hideaki Onishi

**Affiliations:** ^1^Institute for Human Movement and Medical Sciences, Niigata University of Health and Welfare, Niigata, Japan; ^2^Discipline of Physiology, Adelaide Medical School, The University of Adelaide, Adelaide, SA, Australia; ^3^Department of Physical Therapy, Niigata University of Health and Welfare, Niigata, Japan

**Keywords:** brain-derived neurotrophic factor genotype, motor-evoked potential, primary motor cortex, transcranial magnetic stimulation, non-invasive brain stimulation

## Abstract

Techniques of non-invasive brain stimulation (NIBS) of the human primary motor cortex (M1) are widely used in basic and clinical research to induce neural plasticity. The induction of neural plasticity in the M1 may improve motor performance ability in healthy individuals and patients with motor deficit caused by brain disorders. However, several recent studies revealed that various NIBS techniques yield high interindividual variability in the response, and that the brain-derived neurotrophic factor (BDNF) genotype (i.e., Val/Val and Met carrier types) may be a factor contributing to this variability. Here, we conducted a systematic review of all published studies that investigated the effects of the BDNF genotype on various forms of NIBS techniques applied to the human M1. The motor-evoked potential (MEP) amplitudes elicited by single-pulse transcranial magnetic stimulation (TMS), which can evaluate M1 excitability, were investigated as the main outcome. A total of 1,827 articles were identified, of which 17 (facilitatory NIBS protocol, 27 data) and 10 (inhibitory NIBS protocol, 14 data) were included in this review. More than two-thirds of the data (70.4–78.6%) on both NIBS protocols did not show a significant genotype effect of NIBS on MEP changes. Conversely, most of the remaining data revealed that the Val/Val type is likely to yield a greater MEP response after NIBS than the Met carrier type in both NIBS protocols (21.4–25.9%). Finally, to aid future investigation, we discuss the potential effect of the BDNF genotype based on mechanisms and methodological issues.

## Introduction

Non-invasive brain stimulation (NIBS) to the primary motor cortex (M1) induces neural plasticity and is being used ubiquitously as a leading-edge neurorehabilitation tool. Methods of NIBS, including magnetic stimulation or electrical stimulation to the M1 through the scalp, can temporally control M1 excitability toward an increased or decreased value (Nitsche and Paulus, 2000; Di Lazzaro et al., 2010). Various types of NIBS protocols are applied to patients with stroke to promote recovery from motor deficit by modulating abnormal M1 excitability toward the normal direction (Hummel and Cohen, 2006). Although NIBS is widely used as a neurorehabilitation tool, considerable interindividual variability in the response after NIBS has recently been reported (Hamada et al., 2013; López-Alonso et al., 2014; Wiethoff et al., 2014). For instance, only half of the participants showed the expected motor-evoked potential (MEP) changes that express M1 excitability after transcranial direct current stimulation (tDCS) to the M1 (Wiethoff et al., 2014). López-Alonso et al. (2014) investigated the degree of interindividual variability in the response using tDCS, paired-associative stimulation (PAS), and theta burst stimulation (TBS), which were shown to enhance M1 excitability but did not yield the expected MEP changes in 55–61% of the participants. Furthermore, a clinical study reported that motor performance was improved in half of the patients with stroke who had motor deficits after 10 Hz repetitive transcranial magnetic stimulation (rTMS) (Ameli et al., 2009). These studies suggest that considerable interindividual heterogeneity exists in the capacity of individuals to induce M1 plasticity, both in healthy participants and in patients. Therefore, investigation of this issue is necessary to perform effective NIBS and improve motor performance in majority of the participants.

Various factors affect the interindividual variability in MEP changes after NIBS, such as age (Opie et al., 2018), circadian rhythms (Sale et al., 2007), and alpha power oscillations around the M1 (Zrenner et al., 2018). Brain-derived neurotrophic factor (BDNF) genotype may also be one of these factors. Brain-derived neurotrophic factor is a member of the neurotrophin family that is expressed throughout the central nervous system and plays a vital role in synaptic plasticity (Zhou et al., 2004). A single-nucleotide polymorphism that is widely observed at a frequency of 0–72% worldwide (Petryshen et al., 2010) has been identified in the human BDNF gene at codon 66 (Val/66Met), and the replacement of Val66 with Met66 has been reported to reduce BDNF secretion compared with the typical Val/Val type (Chen et al., 2006). Kleim et al. (2006) reported for the first time that the BDNF genotype modulates the M1 plasticity induced by motor training in humans. This study reported that an increase in MEP and expansion of the M1 area after motor training were observed in the Val/Val group, but not in the atypical Val/Met and Met/Met groups. Subsequently, Cheeran et al. (2008) investigated the effect of the BDNF genotype on the MEP changes induced by NIBS and found that the NIBS effects were greater in the Val/Val group than in the Non-Val/Val (Val/Met+Met/Met) group. Therefore, the typical genotype may have a greater effect on NIBS than the atypical genotype, which may contribute to the high interindividual variability in the response regarding MEP changes after NIBS, as mentioned above. However, several subsequent studies using various types of NIBS protocols failed to achieve the same result. For example, Antal et al. (2010) showed that Val/Met had a greater effect on tDCS than did Val/Val, whereas Li Voti et al. (2011) revealed no significant effect on TBS between the genotype groups. The introduction and discussion of several previous articles mentioned the contribution of the BDNF genotype to interindividual variability in the response after NIBS; however, it is still unclear whether the effect of NIBS on the M1 is modulated by the BDNF genotype.

Herein, we conducted a systematic review to investigate the effect of the BDNF genotype on NIBS to the M1. This review classified NIBS intervention with a facilitatory NIBS protocol and an inhibitory NIBS protocol based on MEP changes. Transcranial magnetic stimulation (TMS) measurement with MEP is commonly used to non-invasively assess the M1 excitability changes induced by the NIBS protocol. Although it is still controversial whether the MEP changes induced by NIBS are associated with motor performance, MEP can capture the M1 plasticity changes via comparison of the amplitudes. Thus, we aimed to investigate the functional difference on the M1 plasticity using MEP at a neural level. If the NIBS effect varies depending on the genotype, the secretion of BDNF could contribute to this difference. Therefore, this review also discussed the mechanisms via which the difference in BDNF secretion modulates the M1 plasticity after the NIBS protocol. Furthermore, to aid future investigation, we raised some methodological issues in studies that investigated the effect of the genotype on NIBS based on the assessment of the collected articles.

## Methods

### Protocol

This systematic review was conducted according to the guidelines of the Preferred Reporting Items for Systematic Reviews and Meta-analyses for Protocols 2015 (PRISMA-P 2015) (Moher et al., 2015).

### Eligibility Criteria

Articles were selected if they satisfied the following eligibility criteria: (1) peer-reviewed articles published in English; (2) articles reporting studies performed on healthy adult humans (but not the elderly) not taking any psychoactive medications or drugs; (3) articles that received formal ethical approval; (4) the outcome had to include subjects who were classified into BDNF genotype groups and underwent MEP measurements with single-pulse TMS before and after the NIBS protocol (within 24 h); (5) rTMS, TBS, tDCS, transcranial alternating current stimulation (tACS), transcranial random noise stimulation (tRNS), PAS, and quadri-pulse stimulation (QPS), which can modulate M1 excitability, were selected as a NIBS protocol; (6) the outcomes were obtainable from the main text, figures, tables, or supplementary data; (7) one NIBS protocol was administered to the M1 as an intervention, expect for a combination of NIBS and sham (e.g., rTMS+sham tDCS) or resting condition (rTMS+resting condition); (8) first pre/post measurements were included in cases of a NIBS protocol that aimed at homeostatic plasticity using two interventions at different time points; and (9) TMS was performed on the left M1 or right M1, and MEP was recorded from the contralateral side. The titles and abstracts of the articles were initially screened by one reviewer (R.S). Eligibility of the studies was determined independently by two reviewers (R.S and S.K), who assessed the full text against the inclusion and exclusion criteria.

### Search Strategy and Study Selection

A literature search was conducted using the scientific databases PubMed and Web of Science on April 21, 2021 for articles published from January, 2000 to April 21, 2021. The following search terms were included in these combinations: “transcranial direct current stimulation,” “repetitive transcranial magnetic stimulation,” “theta burst stimulation,” “paired associative stimulation,” “transcranial alternating current stimulation,” “transcranial random noise stimulation,” or “quadri-pulse stimulation” + “brain-derived neurotrophic factor,” “BDNF,” or “Val66Met.” Furthermore, a manual search was carried out over the reference sections of the retrieved studies.

### Data Extraction and Assessment

The MEP amplitudes elicited by single-pulse TMS were investigated as main outcome measures. The Tables 1, 2 that summarized the effect of genotype on the NIBS protocol was divided into two categories, i.e., facilitatory and inhibitory NIBS protocols. The classification was carried out based on the expected MEP changes after the NIBS intervention, regardless of the actual result. Subjects were classified into the Val/Val, Val/Met, and Met/Met groups. If the Val/Met and Met/Met groups were merged as Met carriers, they were represented as the Non-Val/Val group. We assessed MEP changes in the selected articles before and after the NIBS intervention, based on statistical data. *P*-values < 0.05 were taken as a measure of statistical significance. First, we used ANOVA or *t*-test to evaluate the effect of NIBS on MEP changes for each genotype group by comparing the MEP changes between the pre and post data before and after the NIBS condition or NIBS and sham conditions obtained from the results of the selected articles. We established the existence of a facilitatory or inhibitory effect of NIBS for each genotype group if a significant difference was observed by ANOVA or *t*-test. Second, the effect of genotype on NIBS-induced MEP changes was assessed. Most of the selected articles used ANOVA to assess the main effect of time and genotype, as well as the interaction. If the main effect of the genotype group or the interaction was not significant, we judged that no effect of the genotype was observed on MEP changes based on the NIBS protocol, even if there was a trend. If different effects were observed between time points or genotype groups by post hoc analysis after ANOVA, we established that there was a significant difference between the genotype groups. A few articles used *t*-test without ANOVA to assess the effect of the genotype on MEP changes before and after NIBS. In this case, if a different effect was observed between the time points (i.e., pre and post) for each group or between groups, we judged that there was a significant difference.

**Table 1:** Studies using TMS to examine M1 excitability before and after facilitatory NIBS techniques in healthy participants.

**#**	**Study**	**Group**	***N***	**MEP**	**NIBS technique**	**Measurement**	**Compared with the baseline or sham condition**	**Difference between BDNF genotypes**	**Comments**
1	Antal et al. (2010)	Val/Val	10	FDI or ADM	iTBS	MEP	↑	NS	
		Val/Met	5				↑		
2	Cheeran et al. (2008)	Val/Val	9	FDI	iTBS	MEP	↑	SD	
		Non-Val/Val	9				→		
3	Guerra et al. (2020)	Val/Val	15	FDI	iTBS	MEP	→	NS	iTBS+sham tACS condition
		Non-Val/Val	13				→		
4	Lee et al. (2013)	Val/Val	6	FDI	iTBS	MEP	→	NS	RMT_100% intensity for single-pulse MEP
		Val/Met	13				→		
		Met/Met	4				→		
		Val/Val	6	FDI	iTBS	MEP	→	NS	RMT_120% intensity for single-pulse MEP
		Val/Met	13				→		
		Met/Met	4				→		
		Val/Val	6	FDI	iTBS	MEP	→	NS	RMT_140% intensity for single-pulse MEP
		Val/Met	13				→		
		Met/Met	4				→		
5	Li Voti et al. (2011)	Val/Val	14	FDI	iTBS	MEP	↑	NS	
		Val/Met	7				↑		
6	Marsili et al. (2017)	Val/Val	36	FDI	iTBS	MEP	↑↑	SD	
		Non-Val/Val	14				↑		
7	Mastroeni et al. (2013)	Val/Val	15–17	FDI	iTBS	MEP	↑	NS	Intervention: iTBS+iTBS Monophasic TMS for single-pulse MEP
		Val/Met	10–12				↑		
		Val/Val	15–17	FDI	iTBS	MEP	↑	NS	Intervention: iTBS+iTBS Biphasic TMS for single-pulse MEP
		Val/Met	10–12				↑		
1	Antal et al. (2010)	Val/Val	14	FDI or ADM	A-tDCS	MEP	↑	SD	
		Val/Met	10				↑↑		
8	Fujiyama et al. (2014)	Val/Val	11	FCR	A-tDCS	AURC	→	NS	
		Non-Val/Val	5				→		
9	Jonker et al. (2021)	Val/Val	34	FDI	A-tDCS	MEP	→	NS	
		Non-Val/Val	25				→		
10	Strube et al. (2014)	Val/Val	12	FDI	A-tDCS	MEP	↑	NS	
		Non-Val/Val	8				↑		
11	Teo et al. (2014)	Val/Val	19	FDI	A-tDCS	MEP	→	NS	Different statistics were used for the same data
		Val/Met	19				→		
		Met/Met	20				→		
		Val/Val	19	FDI	A-tDCS	MEP	→	NS	
		Non-Val/Val	39				↑		
1	Antal et al. (2010)	Val/Val	21	FDI or ADM	tRNS	MEP	→	NS	
		Val/Met	8				→		
2	Cheeran et al. (2008)	Val/Val	9	APB	PAS	MEP	→	NS	MEPs were measured from APB (target muscle) and ADM (non-target muscle) at the same time
		Non-Val/Val	9				→		
		Val/Val	9	ADM	PAS	MEP	↑	SD	
		Non-Val/Val	9				→		
12	Cirillo et al. (2012)	Val/Val	12	FDI	PAS	MEP (% Max M-wave)	↑	SD	MEPs were measured from FDI (target muscle) and ADM (non-target muscle) at the same time
		Val/Met	10				→		
		Met/Met	7				→		
		Val/Val	12	ADM	PAS	MEP (% Max M-wave)	↑	NS	
		Val/Met	10				↑		
		Met/Met	7				↑		
13	Missitzi et al. (2011)	Val/Val	10	APB	PAS	MEP	↑	SD	
		Non-Val/Val	4				→		
14	Player et al. (2013)	Val/Val	14	FDI	PAS	MEP	↑	NS	ANOVA was performed with healthy and depressed participant groups
		Non-Val/Val	7				↑		
15	Witte et al. (2012)	Val/Val	15	ADM	PAS	MEP	?	NS	
		Non-Val/Val	15				?		
16	Hwang et al. (2015)	Val/Val	12	FDI	rTMS	MEP	↑↑	SD (Val/Val or Val/Met vs. Met/Met)	rTMS with subthreshold intensity
		Val/Met	19				↑↑		
		Met/Met	9				↑		
		Val/Val	12	FDI	rTMS	MEP	↑↑	SD (Val/Val vs. Met/Met)	rTMS with suprathreshold intensity
		Val/Met	19				↑		
		Met/Met	9				↑		
17	Nakamura et al. (2011)	Val/Val	5	FDI	QPS	MEP	↑	NS	
		Non-Val/Val	7				↑		

*The NIBS effects are indicated by the arrow direction based on the statistical data (i.e., facilitatory effect: ↑; no change: →; inhibitory effect: ↓, or Not available: ?). In addition, when either of the genotype groups had a greater effect on the direction of the facilitation, two arrows were added to express this enhanced effect (i.e., ↑↑). ANOVA, analysis of variance; APB, abductor pollicis brevis; ADM, abductor digiti minimi; A-tDCS, anodal transcranial direct current stimulation; AURC, area under the recruitment curve; BDNF, brain-derived neurotrophic factor; FCR, flexor carpi radialis; FDI, first dorsal interosseus; iTBS, intermittent theta burst stimulation; NIBS, non-invasive brain stimulation; Non-Val/Val, Val/Met+Met/Met; NS, not significant; MEP, motor-evoked potential; PAS, paired-associative stimulation; QPS, quadri-pulse transcranial magnetic stimulation; rTMS, repetitive transcranial magnetic stimulation; RMT, resting motor threshold; SD, significant difference; tACS, transcranial alternating current stimulation; tRNS, transcranial random noise stimulation*.

**Table 2:** Studies using TMS to examine M1 excitability before and after inhibitory NIBS techniques in healthy participants.

**#**	**Study**	**Group**	***N***	**MEP**	**NIBS technique**	**Measurement**	**Compared with the baseline or sham condition**	**Difference between BDNF genotypes**	**Comments**
1	Cheeran et al. (2008)	Val/Val	9	FDI	cTBS	MEP	↓	SD	
		Non-Val/Val	9				→		
2	Guerra et al. (2019)	Val/Val	13	FDI	cTBS	MEP	→	NS	cTBS+sham tACS condition
		Non-Val/Val	13				→		
3	Jannati et al. (2017)	Val/Val	12	FDI	cTBS	MEP	?	SD	Only group data was compared by a *t*-test
		Val/Met	6				?		
4	Marsili et al. (2017)	Val/Val	36	FDI	cTBS	MEP	↓↓	SD	
		Non-Val/Val	14				↓		
5	McDonnell et al. (2013)	Val/Val	10	FDI	cTBS	MEP (% Max M-wave)	→	NS	
		Non-Val/Val	15				→		
6	Mastroeni et al. (2013)	Val/Val	15–17	FDI	cTBS	MEP	↓	NS	Intervention: cTBS+cTBS Monophasic TMS for single-pulse MEP
		Val/Met	10–12				↓		
		Val/Val	15–17	FDI	cTBS	MEP	↓	NS	Intervention: cTBS+cTBS Biphasic TMS for single-pulse MEP
		Val/Met	10–12				↓		
		Val/Val	15–17	FDI	cTBS	MEP	↓	NS	Intervention: cTBS+iTBS Monophasic TMS for single-pulse MEP
		Val/Met	10–12				↓		
		Val/Val	15–17	FDI	cTBS	MEP	↓	NS	Intervention: cTBS+iTBS Biphasic TMS for single-pulse MEP
		Val/Met	10–12				↓		
7	Antal et al. (2010)	Val/Val	11	FDI or ADM	C-tDCS	MEP	↓	NS	
		Val/Met	8				↓		
1	Cheeran et al. (2008)	Val/Val	8	FDI	C-tDCS	MEP	↓	NS	
		Non-Val/Val	8				↓		
8	Di Lazzaro et al. (2012)	Val/Val	21	FDI	C-tDCS	MEP	↓	NS	
		Non-Val/Val	8				↓		
9	Strube et al. (2014)	Val/Val	10	FDI	C-tDCS	MEP	↓	NS	
		Non-Val/Val	12				↓		
10	Nakamura et al. (2011)	Val/Val	5	FDI	QPS	MEP	↓	NS	
		Non-Val/Val	7				↓		

*The NIBS effects are indicated by the arrow direction based on the statistical data (i.e., facilitatory effect: ↑; no change: →; inhibitory effect: ↓, or Not available: ?). In addition, when either of the genotype groups had a greater effect on the direction of the inhibition, two arrows were added to express this enhanced effect (i.e., ↓↓). ADM, abductor digiti minimi; BDNF, brain-derived neurotrophic factor; cTBS, continuous theta burst stimulation; C-tDCS, cathodal transcranial direct current stimulation; FDI, first dorsal interosseus; iTBS, intermittent theta burst stimulation; NIBS, non-invasive brain stimulation; Non-Val/Val, Val/Met+Met/Met; NS, not significant; MEP, motor-evoked potential; QPS, quadri-pulse transcranial magnetic stimulation; rTMS, repetitive transcranial magnetic stimulation; SD, significant difference; tACS, transcranial alternating current stimulation*.

### Risk of Bias

The risk of bias was assessed using the Cochrane Collaboration's tool (Higgins et al., 2011). This tool evaluates the risk of selection bias, performance bias, detection bias, attrition bias, reporting bias, and other biases of the individual studies included in this review. The risk of bias was categorized as low, unclear, or high.

## Results

### Selection of Studies

**Figure 1:**
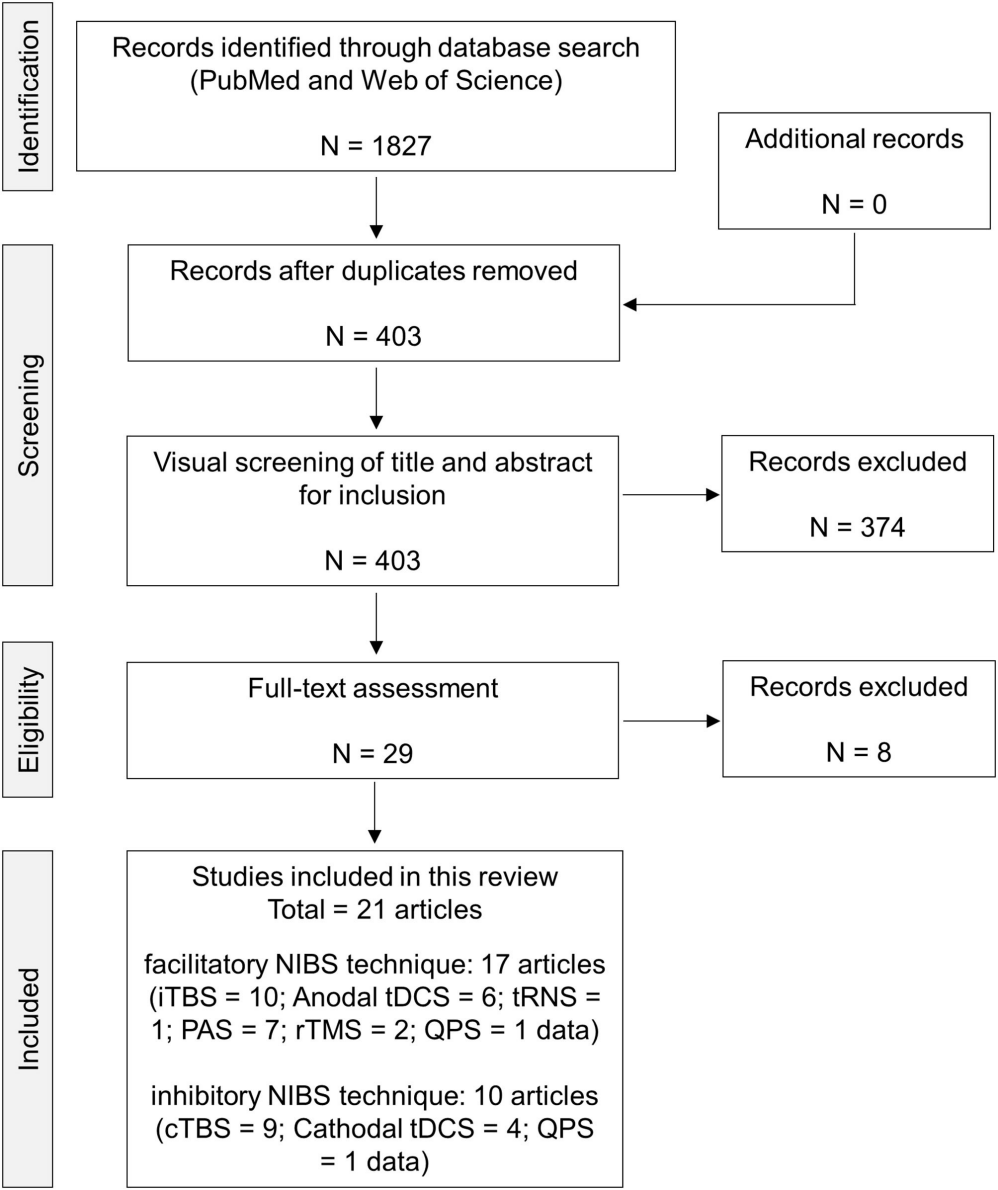
PRISMA flow chart of the present analysis. Here, 1 datum signifies that 1 NIBS intervention and genotype group were included. cTBS, continuous theta burst stimulation; iTBS, intermittent theta burst stimulation; NIBS, non-invasive brain stimulation; PAS, paired-associative stimulation; QPS, quadri-pulse transcranial magnetic stimulation; rTMS, repetitive transcranial magnetic stimulation; tDCS, transcranial direct current stimulation; tRNS, transcranial random noise stimulation.

A flow chart of the current systematic review is presented in Figure 1. Electronic literature searches identified a total of 1,827 studies matching the search terms. The removal of duplicates resulted in 403 studies being retained. An initial screening of the titles and abstracts was performed against the selection criteria, and, in case of insufficient information, full-text articles were scrutinized. The full-text versions of 29 articles were screened for eligibility. A total of eight articles were eliminated by full-text assessment because of the following reasons; (1) single-pulse MEP was not analyzed (Jayasekeran et al., 2011; Myers et al., 2017; Andrews et al., 2020; Jannati et al., 2020); (2) 1 mV MEP was measured before and after the intervention (Deveci et al., 2020); (3) the post MEP measurement was performed after 24 h (Frazer et al., 2016); and (4) healthy adults were not recruited (Jayasekeran et al., 2011; Myers et al., 2017; Jannati et al., 2020). The selected articles were finally categorized into two groups based on the expected results of MEP changes by the NIBS protocol (facilitatory NIBS technique, 17 articles; inhibitory NIBS technique, 10 articles). Regarding the facilitatory protocol, a total of 27 data obtained from 17 articles were extracted: iTBS, 10 data; anodal tDCS, 6 data; tRNS, 1 datum; PAS, 7 data; rTMS, 2 data; and QPS, 1 datum. Regarding the inhibitory protocol, a total of 14 data obtained from 10 articles were extracted; cTBS, 9 data; cathodal tDCS, 4 data; and QPS, 1 datum. Herein, 1 datum means that one NIBS intervention and genotype group were included. Finally, tACS was not identified in both NIBS protocols in the full-text assessment.

### Risk of Bias

**Figure 2:**
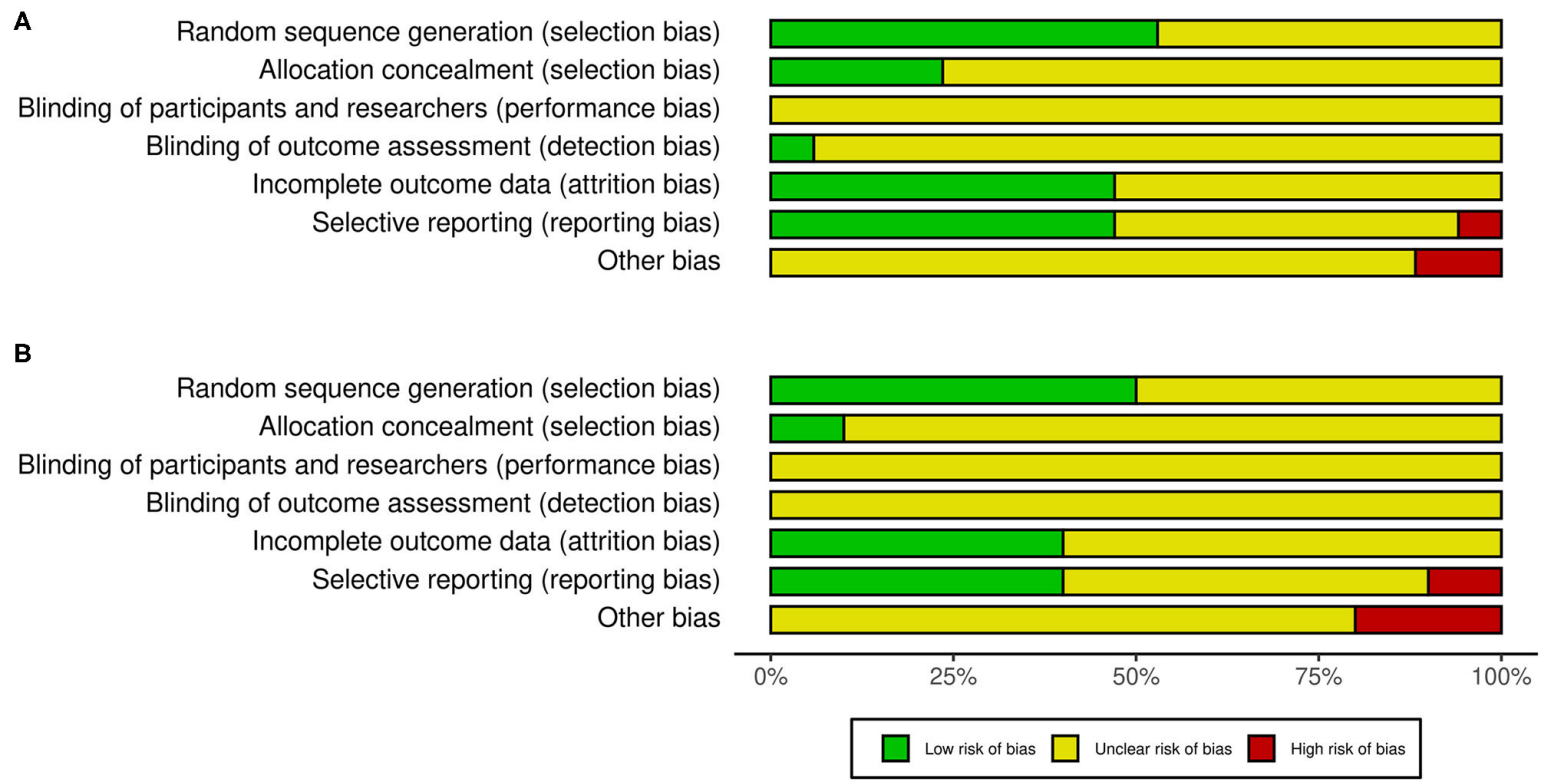
Risk of bias in the studies included in this review. **(A)** Studies with facilitatory NIBS. **(B)** Studies with inhibitory NIBS. NIBS, non-invasive brain stimulation.

The risk of bias of the individual studies included in this review, as judged by the authors, is presented in Figure 2. Generally, the risk of bias varied from low to unclear in all categories. However, 1 out of 17 studies had a high risk for selective bias, and 2 out of 17 studies had a high risk for other biases in the facilitatory NIBS protocol. Conversely, 1 out of 10 studies had a high risk for selective bias, and 2 out of 10 studies had a high risk for other biases in the inhibitory NIBS protocol. The blinding of participants and experimenters was insufficient for BDNF genotyping and NIBS intervention in all studies. However, several studies blinded either BDNF genotyping or NIBS intervention for participants, experimenters, or both (facilitatory NIBS = 7/17 studies; inhibitory NIBS = 3/10 studies). Such studies were classified under unclear risk unless a double-blind design was adopted for both BDNF genotyping and NIBS intervention. Most studies did not include information on the blinding of the outcome assessment.

### Participant and Methodological Characteristics

Tables 1, 2 present a summary of the participant and methodological characteristics of the selected studies. Met carriers were relatively fewer than Val/Val carriers in many studies (facilitatory NIBS: Val/Val = 13.3 ± 7.8 (mean ± SD), Val/Met = 12.0 ± 5.3, Met/Met = 8.0 ± 5.3, Non-Val/Val = 12.6 ± 9.6; inhibitory NIBS: Val/Val = 14.1 ± 9.3, Val/Met = 7.0 ± 1.4, Met/Met = no data, Non-Val/Val = 10.8 ± 3.1). Motor-evoked potentials were recorded from the hand or forearm muscles in all studies. Most studies used MEP amplitude or MEP ratio to assess the genotype effect, whereas one study normalized MEP amplitude to the M-wave from the same muscle [facilitatory NIBS = 1 study (Cirillo et al., 2012); inhibitory NIBS = 1 study (McDonnell et al., 2013)], and another study used the area under the recruitment curve [facilitatory NIBS = 1 study (Fujiyama et al., 2014)].

### Genotype-Related MEP Changes in the Facilitatory NIBS Protocol

**Figure 3:**
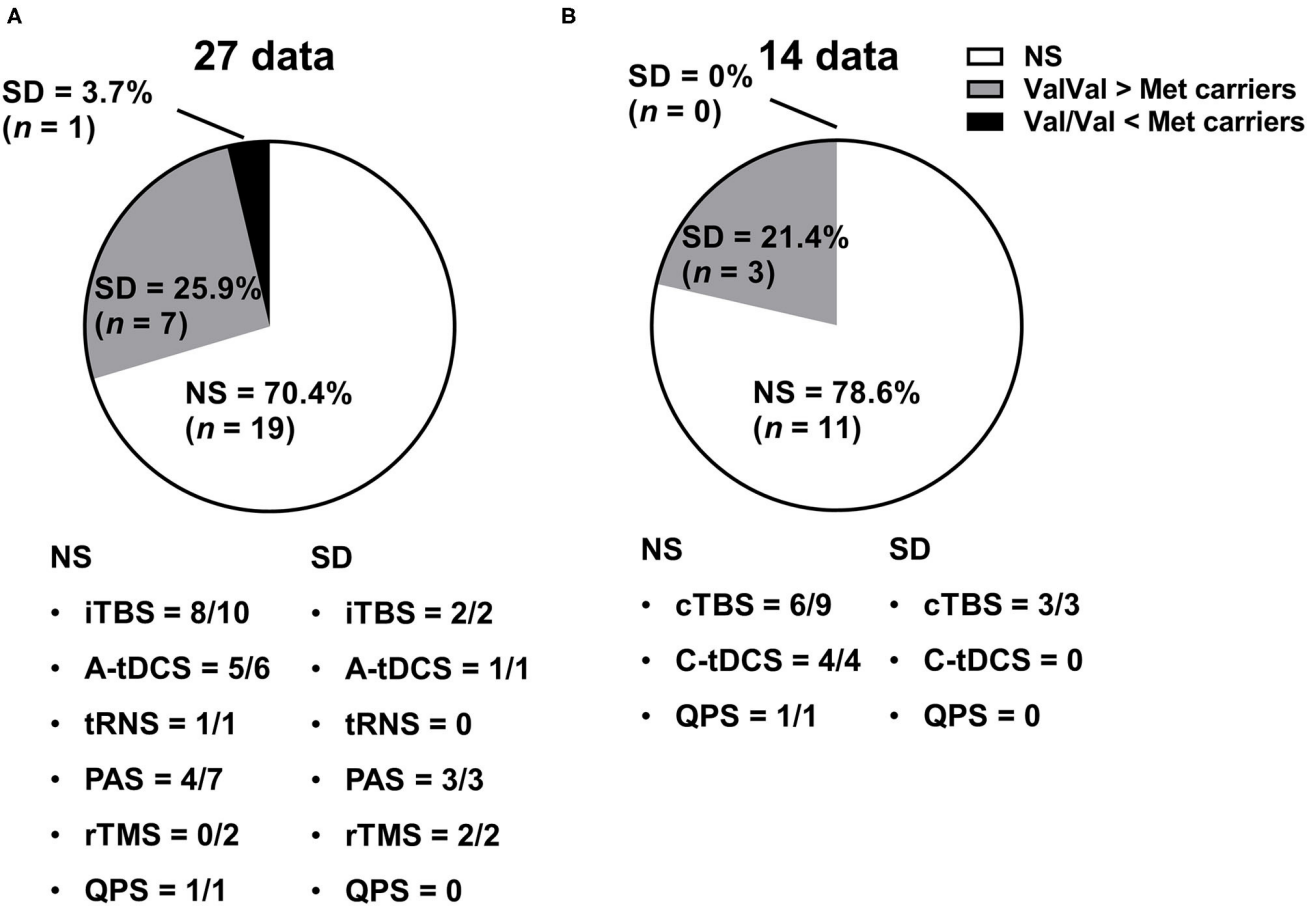
Genetic effect of the NIBS protocol on MEP responses. **(A)** Studies with facilitatory NIBS. **(B)** Studies with inhibitory NIBS. Each pie graph expresses the ratio (%) of how many data showed a significant difference in terms of the BDNF genotype effect. A-tDCS, anodal transcranial direct current stimulation; C-tDCS, cathodal transcranial direct current stimulation; cTBS, continuous theta burst stimulation; iTBS, intermittent theta burst stimulation; NIBS, non-invasive brain stimulation; NS, not significant; PAS, paired-associative stimulation; QPS, quadri-pulse transcranial magnetic stimulation; rTMS, repetitive transcranial magnetic stimulation; SD, significant difference; tRNS, transcranial random noise stimulation.

The studies that reported an effect of genotype on NIBS protocol are illustrated in Figure 3A. In total, 8 of 27 data (29.6%) from 17 studies showed a significant difference between Val/Val and Non-Val/Val (*N* = 4), Val/Met (*N* = 2), or Met/Met (*N* = 3) [1 datum compared between three genotypes, and the result showed that there was a significant difference between Val/Val and Val/Met or Met/Met (Cirillo et al., 2012)]. In general, the expected MEP response after the facilitatory NIBS protocol was greater in the Val/Val than in the Non-Val/Val (*N* = 4), Val/Met (*N* = 1), or Met/Met (*N* = 3) carriers from 7 out of 8 data [1 datum compared between three genotypes, and the result showed that there was a significant difference between Val/Val and Val/Met or Met/Met (Cirillo et al., 2012)], with a significant difference (25.9% in 27 data); however, only 1 out of 8 data indicated Val/Met as having a greater MEP response than Val/Val (3.7% in 27 data) (Antal et al., 2010). Further, 4 out of the 17 studies classified subjects into three BDNF genotype groups (i.e., Val/Val, Val/Met, and Met/Met) (Cirillo et al., 2012; Lee et al., 2013; Teo et al., 2014; Hwang et al., 2015). Also, four studies compared the genotype effect between three genotype groups, but only one out of eight data revealed a significant difference between Val/Met and Met/Met (Hwang et al., 2015). Supplementary data on the assessment of MEP changes before and after the facilitatory NIBS techniques and between BDNF genotypes are presented in Supplementary Table 1.

### Genotype-Related MEP Changes in the Inhibitory NIBS Protocol

Studies that reported the effect of genotype on the NIBS protocol are illustrated in Figure 3B. In total, 3 out of 14 data (21.4%) from 10 studies showed that there was a significant difference between Val/Val and Non-Val/Val (*N* = 2 data), Val/Met (*N* = 1 datum), or Met/Met (*N* = 0 data). The expected inhibitory MEP response after the inhibitory NIBS protocol was greater in the Val/Val than in the Non-Val/Val (*N* = 2 data), Val/Met (*N* = 1 datum), or Met/Met (*N* = 0 data) from three data, with a significant difference (21.4% in 14 data) (Cheeran et al., 2008; Jannati et al., 2017; Marsili et al., 2017); thus, no data showed that Met carriers had a greater inhibitory MEP response than Val/Val carriers (0% in 14 data). None of the studies classified subjects into three BDNF genotype groups (i.e., Val/Val, Val/Met, and Met/Met), which means that there is no report on whether the inhibitory NIBS protocol modulates the MEP response differently between Val/Met and Met/Met carriers. Supplementary data on the assessment of MEP changes before and after the inhibitory NIBS techniques and between BDNF genotypes are presented in Supplementary Table 2.

## Discussion

To the best of our knowledge, this systematic review is the first to investigate whether the BDNF genotype affects the NIBS-induced response to MEPs applied to the M1 of healthy adults. Surprisingly, more than two-thirds of the data on both facilitatory and inhibitory protocols did not show any differences in MEP changes after NIBS between the genotype groups. Regarding the facilitatory protocol, although only 29.6% of the data showed that the MEP response after NIBS varied between the groups, the expected facilitatory MEP response in the Val/Val group was greater than that in the Met carriers in 25.9% of the 27 data. Regarding the inhibitory protocol, 21.4% of the data showed that there is a significant difference on the inhibitory MEP response between genotype groups; however, Val/Val had a greater inhibitory MEP response than did Val/Met among all the data.

A review article investigated the effect of the genotype on the MEP response using iTBS and cTBS (Chung et al., 2016). Although the expected facilitatory iTBS effects were observed in both Val/Val and Met carriers, the long-lasting effects were greater in the Val/Val carriers than in the Met carriers. In contrast, the expected inhibitory cTBS effects were observed in the Val/Val, but not in the Met, carriers. These findings suggest that the BDNF genotype contributes to the MEP modulation after TBS. Conversely, this systematic review summarized the typical NIBS, including TBS, and approximately 20–30% of the data identified genotype-related MEP changes in both facilitatory and inhibitory NIBS protocols. By focusing on the TBS effects, this review revealed that only 2/10 (Cheeran et al., 2008; Marsili et al., 2017) and 3/9 data (Cheeran et al., 2008; Jannati et al., 2017; Marsili et al., 2017) showed genotype-related MEP changes in the facilitatory and inhibitory TBS protocols, respectively; therefore, the genotype effect was not apparent. However, the current and previous reviews were methodologically distinct, involving a different number of selected articles and diverse statistical approaches. Chung et al. (2016) searched articles in 2014, and finally included seven data on iTBS and three data on cTBS in the meta-analysis, which resulted in 3–6 fewer data compared with the current review, which conducted the search in 2021. Furthermore, we defined genotype-related MEP changes after NIBS as those with a significant main effect of the genotype or interaction of the genotype and time upon analysis via ANOVA, although previous several articles used a *t*-test. In contrast, a previous review article normalized MEP data across time data, and subsequently analyzed the data using a simple pre and post comparison in each genotype group. Taken together, the findings of the current review, which judged the significant difference based on the results of ANOVA including multiple main factors, might be more conservative for *P*-value calculation than those of the previous reviews.

Although the genotype effects on MEP changes after NIBS were limited in both NIBS protocols, we discussed the possible mechanisms that produce the differences in MEP changes between genotypes, as described below. Chen et al. (2006) classified mice into Val/Val, Val/Met, and Met/Met groups, and they subsequently measured BDNF secretion in hippocampal cortical neurons. Consequently, BDNF secretion in the Val/Met and Met/Met groups was significantly reduced by 18 and 29%, respectively, suggesting that the BDNF genotype influences BDNF secretion, implying the modulation of brain function in terms of neural plasticity. Several studies also investigated how BDNF regulates brain function in mice and showed that NMDA-receptor-dependent synaptic plasticity was impaired in the hippocampus of Met/Met mice (Ninan et al., 2010), and decreased NMDA and GABA receptor-mediated synaptic transmissions were observed in the pyramidal neurons of the prefrontal cortex in Met/Met mice (Pattwell et al., 2012). Similarly, lower glutamate plus glutamine concentrations, known as an excitatory neurometabolite index, in the M1, as measured by magnetic resonance spectroscopy, were observed in the Non-Val/Val human adults compared with the Val/Val human adults in a previous study (Sasaki et al., 2021). Considering that the glutamatergic and GABAergic activities in the M1 play an important role in the synaptic changes caused by NIBS (Liebetanz et al., 2002; Nowak et al., 2017; Bachtiar et al., 2018; Wischnewski et al., 2019), the decreased BDNF secretion in the Met carriers may weaken M1 plasticity function via the NMDA- and GABA-receptor-dependent activities, consequently producing smaller MEP changes after NIBS than Val/Val carriers. Taken together, these findings suggest that the weaker MEP changes observed in Met carriers after the facilitatory and inhibitory NIBS protocols (21.4–29.6% in all data) in this review are derived from decreased BDNF secretion. Furthermore, as for the predominant NIBS effect in the Val/Val carriers, short-term memory and cognitive functions were greater in the Val/Val carriers than in the Met carriers (Dempster et al., 2005; Ho et al., 2006; Huang et al., 2014).

Met/Met might have a greater impact on brain function than Val/Met in terms of neural activity and BDNF secretion. First, synaptic transmissions via GABA and NMDA receptors were significantly different between Val/Val and Met/Met mice (Ninan et al., 2010; Pattwell et al., 2012). Second, BDNF secretion in the Met/Met mice was less than that in the Val/Met mice (Chen et al., 2006). Despite these findings, majority of the selected articles in the current review merged Val/Met and Met/Met into Met carriers or did not recruit Met/Met participants because, compared with other genotypes, Met/Met carriers in the human population are fewer (Caucasian population: approximately 1–8%) (Shen et al., 2018). Because of this, most studies could not investigate the genotype effect in detail. Conversely, the recruitment of Met/Met participants may be easier in the Asian population owing to the higher prevalence of the Met/Met genotype in this population (approximately 15–23%) (Shen et al., 2018).

Few of the selected articles classified participants into three genotype groups in the facilitatory NIBS protocol. High-frequency rTMS, which induces MEP facilitation, had a poor facilitatory effect in the Met/Met group compared with Val/Val and Val/Met groups (Hwang et al., 2015), whereas the other studies failed to report similar results (Cirillo et al., 2012; Lee et al., 2013; Teo et al., 2014), suggesting that a definitive conclusion could not be reached. These studies included a relatively small sample size for Met/Met participants (*N* = 4–20). Given the genotype-related functional changes in the brain between Val/Met and Met/Met carriers, in addition to the small sample size, future studies including the three groups and a large sample population are required. None of studies reporting inhibitory NIBS data classified participants into three groups.

Cortical volume may also explain the genotype-related MEP changes that occur after NIBS. Neuroimaging studies reported that the cortical volume varied in some cortical regions between the genotype groups (Yang et al., 2012; Jasińska et al., 2017; Shen et al., 2020). When the interaction of cortical volume with the NIBS effect was considered, the volume of the sensorimotor cortex positively predicted the NIBS effect on MEP changes (Conde et al., 2012). However, this hypothesis lacks reliable information, because the different cortical volumes did not correspond to the same regions (Yang et al., 2012; Jasińska et al., 2017; Shen et al., 2020), and some studies failed to show the genotype-related cortical volume changes (Kim et al., 2015; McKay et al., 2019). Moreover, one study showed that the M1 volume in the Met/Met group was larger than that in the other groups (Jasińska et al., 2017).

Although NIBS is becoming a popular rehabilitation tool that modifies M1 excitability in patients with stroke, considerable interindividual variability in MEP changes has been recently reported in healthy participants (Hamada et al., 2013; López-Alonso et al., 2014; Wiethoff et al., 2014). We expected the BDNF genotype to partially contribute to the variability, but found the genotype effect to be relatively small in this review. Therefore, rather than genetic factors, other factors, such as age (Opie et al., 2018), attention (Stefan et al., 2004), time (Sale et al., 2007), and neural oscillations (Zrenner et al., 2018) may contribute more to the variability. For example, tDCS applied to the M1 was less effective in older adults than in young adults (Ghasemian-Shirvan et al., 2020). Furthermore, PAS effect was more pronounced in the afternoon session than in the morning one (Sale et al., 2007). Altogether, a variety of factors may complicatedly interact with each other to affect M1 plasticity.

The differences related to the genotype changes between the facilitatory and inhibitory NIBS protocols are likely to be small. A total of 27 data were collected in the facilitatory NIBS protocol, and a total of 14 data were collected in the inhibitory NIBS protocol. Thus, the number of data was considerably different between these NIBS protocols. Furthermore, majority of the inhibitory NIBS protocols used cTBS (9/14 data), suggesting that tDCS, rTMS, and PAS were performed less frequently in the inhibitory NIBS protocol (tDCS = 3 data; rTMS = 0 data; PAS = 0 data) than in the facilitatory NIBS protocol (tDCS = 6 data; rTMS = 2 data; PAS = 7 data). Taken together, to investigate the genotype-related changes in detail, further studies investigating a variety of NIBS protocols are warranted.

We assessed the risk of bias based on Cochrane Collaboration's tool (Higgins et al., 2011), and found that majority of the bias types had low risk or unclear risk. In most of the selected articles, the performance bias showed an unclear risk, indicating that the blinding of the data collection and analysis were unclear. An epidemiological study reported that incomplete blinding may exaggerate the effect size by approximately 25% (Wood et al., 2008). To improve the quality for this kind of study regarding the BDNF genotype, a double-blind protocol is required for both multiple NIBS interventions and data analysis in BDNF genotype groups. None of the selected articles adopted a double-blind design in both settings, but some adopted a single-blind design for NIBS interventions or BDNF genotyping. Furthermore, the inclusion of a sham NIBS group is desirable in a double-blind protocol to confirm the cumulative effect of single-pulse TMS, which increases the MEP amplitude (Julkunen et al., 2012; Pellicciari et al., 2016).

There are some limitations of the current review. First, we could not investigate the genotype-related NIBS response in detail because of the different genotyping groups. Some classified participants as Val/Val, and Val/Met, and Met/Met, whereas the others merged Val/Met and Met/Met into Non-Val/Val. This difference may affect the judgment of the genotype-related changes in this review. Second, it is unclear whether the genotype-related NIBS effect on MEP changes modulates actual motor performance through MEP changes because recent studies reported that there was no correlation between MEP and motor behavior changes induced by NIBS (López-Alonso et al., 2015; Lopez-Alonso et al., 2018). Although some studies investigated the effect of the genotype on motor performance, similar results were not obtained (McHughen et al., 2010; Li Voti et al., 2011; van der Vliet et al., 2018). Therefore, assessing motor performance, in addition to measuring MEP, may be better for understanding the mechanism at neural and behavioral levels. Finally, we summarized a variety of NIBS protocols to determine the effect of the BDNF genotype across typical NIBS protocols; thus, each NIBS effect was not individually evaluated. Recent studies reported that QPS showed stronger M1 plasticity than TBS (Tiksnadi et al., 2020) and relatively low interindividual variability (Nakamura et al., 2016; Tiksnadi et al., 2020). Furthermore, QPS effect was not influenced by BDNF polymorphism (Nakamura et al., 2011). Therefore, the selection of NIBS from a variety of its protocols may be important to minimize the interindividual variability, but further research is required to determine which NIBS protocol is the most effective inducing M1 plasticity.

## Conclusion

This systematic review investigated whether the BDNF genotype influences MEP modulation after NIBS to the M1 in healthy adults. Our findings revealed that only approximately 20–30% of the selected data showed BDNF-genotype-dependent changes in the MEP response, suggesting that the genotype effect may have a lesser impact than previously anticipated. However, because majority of the articles merged Val/Met and Met/Met into Met carriers, future studies classifying the participants into three groups are required for both facilitatory and inhibitory NIBS protocols. Although the genotype-related MEP changes detected after NIBS were relatively small, Val/Val is likely to have a greater effect on NIBS than Met carriers for both NIBS protocols. This difference may be associated with the decreased BDNF secretion in Met carriers, which results in the poor induction of M1 plasticity.

## Data Availability Statement

The original contributions presented in the study are included in the article/Supplementary Material, further inquiries can be directed to the corresponding author/s.

## Author Contributions

HO and RS conceived the study, designed the experiments, and wrote the manuscript. RS and SK reviewed the articles and performed data extraction and analysis. All authors read and approved the final manuscript.

## Funding

This work was supported by a Grant-in-aid for Scientific Research (A) from the Japan Society for the Promotion of Science (grant number 19H01090) and Overseas Research Fellowship from the Japan Society for the Promotion of Science (grant number: 202060103).

## Conflict of Interest

The authors declare that the research was conducted in the absence of any commercial or financial relationships that could be construed as a potential conflict of interest.

## Publisher's Note

All claims expressed in this article are solely those of the authors and do not necessarily represent those of their affiliated organizations, or those of the publisher, the editors and the reviewers. Any product that may be evaluated in this article, or claim that may be made by its manufacturer, is not guaranteed or endorsed by the publisher.
