# Supplementary material for: Do Brain-Derived Neurotrophic Factor Genetic Polymorphisms Modulate the Efficacy of Motor Cortex Plasticity Induced by Non-invasive Brain Stimulation? A Systematic Review
Source: Front Hum Neurosci. 2021 Sep 28;15:742373. doi: 10.3389/fnhum.2021.742373 (PMC8505675; doi:10.3389/fnhum.2021.742373)
Supplement: Supplementary file 1 [file Data_Sheet_1.pdf]

Supplementary Data

Supplementary table 1. Support for judgement on MEP changes before and after facilitatory NIBS techniques and between BDNF genotypes in healthy participants.

#	Study	Group	NIBS technique	MEP changes	Support for judgement on MEP changes before and after NIBS	Genotype effect	Support for judgement on MEP changes between genotypes
1	Antal et al. (2010)	Val/Val<br>Val/Met	iTBS	↑<br>↑	ANOVA of the normalized data revealed a significant main effect of TIME	NS	Although authors said that excitability enhancement was more pronounced in the Val/Met group, the Genotype * Time interaction was not significant
2	Cheeran et al. (2008)	Val/Val<br>Non-Val/Val	iTBS	↑<br>→	There was a significant increase in MEPs after iTBS in the Val/Val but not in the non-Val/Val	SD	There was a significant increase in MEP after iTBS in Val/Val but not in the non-Val/Val group
3	Guerra et al. (2020)	Val/Val<br>Non-Val/Val	iTBS	→<br>→	From Fig. 2 in the article	NS	From Fig. 2 in the article
4	Lee et al. (2013)	Val/Val<br>Val/Met<br>Met/Met	iTBS	→<br>→<br>→	Results showed the increases in MEP were statistically significant in Val/Val, but not in the other groups at 100% RMT by t-test. Moreover, at 120 and 140% intensities, MEP increased significantly in Val/Val and Val/Met. However, authors said that the iTBS paradigm tended to increase MEP at all the three stimulus intensities regardless of genotypes before t-test, and thus it would probably mean there was no significant time	NS	Authors said that the iTBS paradigm tended to increase MEP at all the three stimulus intensities (100, 120, and 140% RMT) regardless of genotypes. This statement would mean there were no significant results through ANOVA, but ANOVA results were not listed. When we looked Fig. 2C, different MEP changes were observed between

					effect using ANOVA. We finally judged there was no significant time effect for each TMS intensity condition.		genotype groups by t-test. However, we judged there was no significant difference between groups based on the above ANOVA results.
		Val/Val		→			
		Val/Met	iTBS	→		NS	
		Met/Met		→			
		Val/Val		→			
		Val/Met	iTBS	→		NS	
		Met/Met		→			
5	Li Volti et al. (2011)	Val/Val	iTBS	↑	ANOVA showed a significant main effect of time, no significant effect of Group and interaction	NS	Between group ANOVA for MEP size after iTBS showed a no significant effect of Group and no significant interaction
		Val/Met		↑			
6	Marsili et al. (2017)	Val/Val	iTBS	↑ ↑	ANOVAs showed a significant main effect of time (iTBS: $P < 0.001$)	SD	iTBS and induced greater changes in Val/Val compared to Met carriers at T5, T15, and T30
		Non-Val/Val		↑			
7		Val/Val	iTBS	↑		NS	ANOVAs for monophasic MEP revealed no impact of polymorphism

		Val/Met		↑	One sampled t-tests confirmed that iTBS consistently induced an increase in monophasic MEP		
	Mastroeni et al. (2013)	Val/Val	iTBS	↑	One sampled t-tests confirmed that iTBS consistently induced an increase in monophasic MEP	NS	ANOVAs for biphasic MEP revealed no impact of polymorphism
		Val/Met		↑			
1	Antal et al. (2010)	Val/Val	A-tDCS	↑	There was a significant increase in MEPs after A-tDCS in both groups	SD	The enhancement was more pronounced in Val/Met (Val/Val: T20, *P* < 0.03; Val/Met: T0-T60, *P* < 0.01)
		Val/Met		↑↑			
8	Fujiyama et al. (2014)	Val/Val	A-tDCS	→	All main effects and interactions including Sex and/or BDNF as a factor were not statistically significant	NS	All main effects and interactions including Sex and/or BDNF as a factor were not statistically significant
		Non-Val/Val		→			
9	Jonker et al. (2021)	Val/Val	A-tDCS	→	None of the models showed a main effect of tDCS on cortical excitability	NS	The BDNF model did not show an interaction effect of the BDNF genotype
		Non-Val/Val		→			
10	Strube et al. (2015)	Val/Val	A-tDCS	↑	MEP increased in both groups following A-tDCS, demonstrated by a significant main effect for time	NS	There was no significant difference between genotype groups by t-test
		Non-Val/Val		↑			

11	Teo et al. (2014)	Val/Val	A-tDCS	→	ANOVA using the between-subject factor 'genotype' and within-subject factor 'time' showed no effect of 'genotype', 'time' or interaction	NS	ANOVA using the between-subject factor 'genotype' and within-subject factor 'time' showed no effect of 'genotype', 'time' or interaction
		Val/Met		→			
		Met/Met		→			
		Val/Val	A-tDCS	→	Met carriers showed a significantly increased cortical excitability after A-tDCS	NS	ANOVA showed a significantly increased MEP for Met carriers after A-tDCS, but no difference between groups
		Non-Val/Val		↑			
1	Antal et al. (2010)	Val/Val	tRNS	→	ANOVA of the normalized data revealed no significant main effect of TIME	NS	ANOVA of the normalized data revealed no significant main effect of Genotype, and the interaction
		Val/Met		→			
2	Cheeran et al. (2008)	Val/Val	PAS	→	In Val/Val, PAS produced a borderline significant increase in APB (*P* = 0.07) but not in the non-Val/Val	NS	In Val/Val, PAS produced a borderline significant increase in APB (*P* = 0.07) but not in non-Val/Val
		Non-Val/Val		→			
		Val/Val	PAS	↑	In Val/Val, PAS produced a significant increase of the MEPs in ADM but not in non-Val/Val	SD	In Val/Val, PAS produced a significant increase of the MEP in ADM but not in non-Val/Val
		Non-Val/Val		→			

12	Cirillo et al. (2012)	Val/Val		↑			
		Val/Met	PAS	→	FDI MEP was significantly greater in the Val⁄Val after PAS, but there was no effect of PAS in the Val⁄Met or Met⁄Met	SD	FDI MEP amplitude was significantly greater in the Val⁄Val after PAS, but there was no effect of PAS in the Val⁄Met or Met⁄Met
		Met/Met		→			
		Val/Val		↑			
		Val/Met	PAS	↑	For the non-target ADM, there was a significant increase in MEP, but no difference between groups and no interaction	NS	For the non-target ADM, there was a significant increase in MEP for all subjects combined (time effect), but no difference between groups and no interaction
		Met/Met		↑			
13	Missitzi et al. (2011)	Val/Val		↑			
			PAS		In Met alleles, PAS led to no enhancement of MEP. In contrast, MEP was enhanced in Val/Val.	SD	In Met alleles, PAS led to no enhancement of MEP. In contrast, MEP was enhanced in Val/Val.
		Non-Val/Val		→			
14	Player et al. (2013)	Val/Val		↑			
			PAS		Tests of simple effects found a significant increase in the MEP after PAS for the healthy group	NS	There was no significant effect of genotype, and no group (healthy vs. depressed patients)–genotype interaction
		Non-Val/Val		↑			
15	Witte et al. (2012)	Val/Val					
			PAS	?	Not listed	NS	There was no significant difference between genotype groups by t-test
		Non-Val/Val					
16	Hwang et al. (2015)	Val/Val		↑↑			
			rTMS		From Fig. 2 in the article	SD	From Fig. 2 in the article (rTMS with subthreshold intensity)
		Val/Met		↑↑			

		Met/Met		↑			
		Val/Val		↑↑			
		Val/Met	rTMS	↑	From Fig. 2 in the article	SD	From Fig. 2 in the article (rTMS with suprathreshold intensity)
		Met/Met		↑			
17	Nakamura et al. (2011)	Val/Val	QPS	↑	Post hoc analyses revealed that significant potentiation was elicited by QPS	NS	ANOVA revealed no significant effect of Group, and no significant interaction (Group * Time)
		Non-Val/Val		↑			

Abbreviations: ADM, abductor digiti minimi; ANOVA, analysis of variance; A-tDCS, anodal transcranial direct current stimulation; BDNF, brain-derived neurotrophic factor; FDI, first dorsal interosseus; iTBS, intermittent theta burst stimulation; NIBS, noninvasive brain stimulation; Non-Val/Val, Val/Met+Met/Met; NS, not significant; MEP, motor-evoked potential; PAS, paired associative stimulation; QPS, quadripulse transcranial magnetic stimulation; rTMS, repetitive transcranial magnetic stimulation; RMT, resting motor threshold; SD, significant difference; T, timepoint; tACS, transcranial alternating current stimulation; tRNS, transcranial random noise stimulation.

Supplementary table 2. Support for judgement on MEP changes before and after inhibitory NIBS techniques and between BDNF genotypes in healthy participants.

#	Study	Group	NIBS technique	MEP changes	Support for judgement on MEP changes before and after NIBS	Genotype effect	Support for judgement on MEP changes between BDNF genotypes
1	Cheeran et al. (2008)	Val/Val	cTBS	↓	There was a significant decrease in MEP after cTBS in Val/Val but not in non-Val/Val	SD	There was a significant decrease in MEPs after cTBS in Val/Val but not in non-Val/Val group
		Non-Val/Val		→			
2	Guerra et al. (2020)	Val/Val	cTBS	→	From Fig. 3 in the article	NS	From Fig. 3 in the article
		Non-Val/Val		→			
3	Jannati et al. (2017)	Val/Val	cTBS	?	Only BDNF genotype groups were compared by t-test	SD	MEP at T10 was significantly reduced in BDNF Val/Met participants than in BDNF Val/Val participants
		Val/Met		?			
4	Marsili et al. (2017)	Val/Val	cTBS	↓ ↓	ANOVAs showed a significant main effect of time (cTBS: P < 0.001)	SD	cTBS and induced greater changes in Val/Val compared to Met carriers at T5, T15, and T30
		Non-Val/Val		↓			
5	McDonnell et al. (2013)	Val/Val	cTBS	→	From Fig. 5 in the article	NS	The mixed-model analysis did not reveal a main effect of genotype, nor any genotype * time, genotype * condition, or genotype * condition* time interactions
		Non-Val/Val		→			

6	Mastroeni et al. (2013)	Val/Val<br>Val/Met	cTBS	↓<br>↓	One sampled t-test confirmed that cTBS consistently induced a decrease in monophasic MEP	NS	ANOVA for monophasic MEP revealed no impact of polymorphism
		Val/Val<br>Val/Met	cTBS	↓<br>↓	One sampled t-test confirmed that cTBS consistently induced a decrease in biphasic MEP	NS	ANOVA for biphasic MEP revealed no impact of polymorphism
		Val/Val<br>Val/Met	cTBS	↓<br>↓	One sampled t-test confirmed that cTBS consistently induced a decrease in monophasic MEP	NS	ANOVA for monophasic MEP revealed no impact of polymorphism
		Val/Val<br>Val/Met	cTBS	↓<br>↓	One sampled t-tests confirmed that cTBS consistently induced a decrease in biphasic MEP	NS	ANOVA for biphasic MEP revealed no impact of polymorphism
7	Antal et al. (2010)	Val/Val<br>Val/Met	C-tDCS	↓<br>↓	There was a significant decrease in MEP after C-tDCS in both genotype groups	NS	Two-way ANOVA revealed no significant main effect of Genotype; however, the Time was significant. The interaction was not significant.
		Val/Val	C-tDCS	↓		NS	From Fig. 2 in the article

	Cheeran et al. (2008)	Non-Val/Val		↓	Val/Val and non-Val/Val showed the expected pattern of effects: C-tDCS suppressed MEP		
8	Di Lazzaro et al. (2012)	Val/Val	C-tDCS	↓	Post hoc test showed a significant reduction of MEP immediately after stimulation	NS	The BDNF genotype was not significant
		Non-Val/Val		↓			
9	Strube et al. (2015)	Val/Val	C-tDCS	↓	Plasticity response was observed in Val/Val and Non-Val/Val	NS	Independent-sample t-tests revealed no significant differences between genotype groups
		Non-Val/Val		↓			
10	Nakamura et al. (2011)	Val/Val	QPS	↓	Post hoc analyses showed significant suppression by QPS	NS	ANOVA revealed no significant effect of Group, and no significant interaction (group * time)
		Non-Val/Val		↓			

Abbreviations: cTBS, continuous theta burst stimulation; C-tDCS, cathodal transcranial direct current stimulation; iTBS, intermittent theta burst stimulation; NIBS, noninvasive brain stimulation; Non-Val/Val, Val/Met+Met/Met; NS, not significant; MEP, motor-evoked potential; QPS, quadripulse transcranial magnetic stimulation; rTMS, repetitive transcranial magnetic stimulation; SD, significant difference; T, timepoint; tACS, transcranial alternating current stimulation.
